# Delayed antibiotic prescription for upper respiratory tract infections in children under primary care: Physicians’ views

**DOI:** 10.1080/13814788.2017.1347628

**Published:** 2017-07-17

**Authors:** Camilla Flintholm Raft, Lars Bjerrum, Magnus Arpi, Jens Otto Jarløv, Jette Nygaard Jensen

**Affiliations:** ^a^ Department of Clinical Microbiology, Herlev and Gentofte Hospital, University of CopenhagenHerlevDenmark; ^b^ Danish Patient Safety AuthorityCopenhagenDenmark; ^c^ Department of Public Health, Section of General Practice, University of CopenhagenCopenhagenDenmark

**Keywords:** General practice, antibacterial agents, antibiotic prescription, children, respiratory tract infections

## Abstract

**Background:** Overprescribing antibiotics for common or inaccurately diagnosed childhood infections is a frequent problem in primary healthcare in most countries. Delayed antibiotic prescriptions have been shown to reduce the use of antibiotics in primary healthcare.

**Objective:** The aim was to examine primary care physicians’ views on delayed antibiotic prescriptions to preschool children with symptoms of upper respiratory tract infections (URTIs).

**Methods:** A questionnaire was sent to 1180 physicians working in general practice in the Capital Region of Denmark, between January and March 2015. The questions focused on physicians’ attitude and use of delayed antibiotic prescriptions to children with URTIs.

**Results:** The response rate was 49% (*n* = 574). Seven per cent of the physicians often used delayed prescriptions to children with symptoms of URTI, but 46% believed that delayed prescription could reduce antibiotic use. The physicians’ views on delayed antibiotic prescription were significantly associated with their number of years working in general practice. Parents’ willingness to wait-and-see, need for reassurance, and knowledge about antibiotics influenced the physicians’ views. Also, clinical symptoms and signs, parents’ willingness to shoulder the responsibility, the capability of observation without antibiotic treatment, and structural factors like out-of-hour services were relevant factors in the decision.

**Conclusions:** Most physicians, especially those with fewer years of practice, had a positive attitude towards delayed antibiotic prescription. Several factors influence the views of the physicians—from perceptions of parents to larger structural elements and years of experience.

KEY MESSAGES

- Delayed antibiotic prescriptions reduce the use of antibiotics.
- Most responding physicians—especially those with fewer years of experience in primary care—were positive towards delayed antibiotic prescription.

## Introduction

Antibiotic resistance is a growing public health concern and closely related to use of antibiotics [1]. In Denmark, 90% of prescriptions for antibiotics are issued in primary healthcare, and the majority are for respiratory tract infections [2,3]. Most upper respiratory tract infections (URTIs) are self-limiting and caused by viruses, on which antibiotics have no effect [4,5]. Studies have shown that a significant number of antibiotic prescriptions are unnecessary and even harmful due to their adverse effects [4,6].

A substantial variation in antibiotic prescription among children across European countries suggests that antibiotics have been inappropriately used [7]. The high and unnecessary use of antibiotics is especially common among preschool children [8]. A study has shown that the number of antibiotic prescriptions to Danish children has been high, but stable, during 2000–2012. Of the five regions in Denmark, the Capital Region has the highest antibiotic consumption [9].

Delayed prescription is one of the best strategies available to reduce the use of antibiotics, without influencing patient morbidity or mortality [10,11]. Delayed antibiotic prescription means that the patient is given a prescription and told to wait-and-see if the symptoms disappear spontaneously. If the symptoms worsen, the prescription should be redeemed. The prescription is generally valid for a week [12].

Delayed antibiotic prescription was officially introduced in Denmark in 2014 through the Danish College of General Practitioners’ clinical guidelines on RTI. It has been recommended to consider delayed prescription in URTIs with a high degree of self-limitation [12]. In 2008, the UK National Institute for Health and Care Excellence published guidelines for implementing alternatives to an immediate antibiotic prescription, including the delayed antibiotic prescription [13].

While the positive effects of delayed antibiotic prescription are well documented, little is known about the physicians’ views on delayed antibiotic prescription. Denmark is among the countries with the lowest prescription rates among children in Europe [7]. To reduce the high and unnecessary use of antibiotics, a deeper insight into physicians’ views about delayed prescription is important.

In Denmark, general physicians are responsible for the primary care of the whole population, including children. This study sought to examine general physicians’ views on delayed antibiotic prescription in preschool children with symptoms of URTIs.

## Methods

### Study design and subjects

A questionnaire was developed and distributed to 1105 general practitioners (GPs), including 75 physicians in training, in Denmark between January and March 2015.

The addresses of the GPs were identified via MedCom.dk, while the names of the physicians were identified via Sundhed.dk. The physicians received the questionnaire by post. A stamped self-addressed envelope was enclosed for reply. About ten days after the questionnaires were sent; the physicians who did not respond received a reminder by post.

The questionnaire sought to collect demographic details such as gender, practice location, type of physician (GP or trainee), and the number of years in general practice, and practice type (single or partnership). Questions regarding delayed antibiotic prescription were also included:

- How often do you use delayed antibiotic prescription in children with symptoms of URTIs?
- Do you believe delayed antibiotic prescription can reduce the use of antibiotics in children with URTIs?
- Why or why not? (Open text field.)

The provision of an open text field made it possible for the physicians to elaborate their views on delayed antibiotic prescription. A definition of delayed antibiotic prescription preceded the questions: ‘A “wait and see” prescription is a time-limited antibiotic prescription. If symptoms continue, the patient can redeem the prescription.’ The questionnaire also included five questions about the GPs’ perceptions of parental knowledge and expectations, which are not reproduced in this article.

### Validity of the questionnaire

To maximize its validity, the questionnaire was pretested on relevant respondents before distribution; two GPs filled the questionnaire as a pilot study, and in-depth cognitive interviews were carried out to examine how the physicians understood and responded to the questions [14]. In addition, two experts in the field of survey design approved the quality of the questionnaire. After the pretest, adjustments in phrasings were made, and an additional question was included.

### Statistical and qualitative analyses

Logistic regression analyses were performed with the dependent variable being whether the physicians believed delayed antibiotic prescription could reduce the number of antibiotics used to treat URTIs in preschool children. The respondents who replied ‘no’ or ‘don’t know’ were grouped together. Excluding those respondents who replied ‘don’t know’ from analyses did not affect the results of the survey. Analyses were performed using SPSS Statistics 22.

We used an inductive approach to analyse the qualitative data. The comments from the open text field were coded to discover patterns in the comments and then categorized in main and subtopics. In this process, efforts have been made to eliminate the influence of the pre-understanding of the researcher, and the topics were defined by data allowing an open-minded analysis of the qualitative data.

## Results

Out of a total of 1180 physicians, 574 responded (49%). Of these, 322 used the open text field to elaborate their views. Table 1 shows the characteristics of physicians who responded. No evidence of bias was found when comparing responding physicians with non-responding physicians on the following characteristics: type of physician (GP or trainee), gender, practice location, and level of antibiotic use in the physician’s municipality.

**Table 1.** Characteristics of responding physicians.

	*n* (%)
Type of physician	
GP	533 (93)
GP trainee	41 (7)
Gender	
Female	313 (55)
Male	261 (45)
Number of years working in general practice	
0–5 (including trainees)	125 (22)
6–14	169 (29)
15–24	163 (28)
25 or more	117 (20)
Practice location	
Copenhagen	198 (35)
Suburban municipalities	163 (28)
Bornholm Island	20 (4)
North Zealand	188 (33)
Antibiotic use in municipality	
Lower than regional mean^a^	416 (73)
Higher than regional mean^a^	153 (27)

^a^739 prescriptions per 1000 0–6-year-old children in Copenhagen in 2012.

### Delayed prescription—how often?

Of the respondents, 21% never issued a delayed antibiotic prescription to children with symptoms of URTIs, 40% rarely did so while 32% did it sometimes, and 7% often used delayed prescriptions. A physician’s use of delayed prescription was not significantly related to gender or number of years in practice, the location of practice, or level of antibiotic use in the physician’s municipality.

### Views on delayed prescription

Almost half of the respondents (46%) were positive about the potential of delayed antibiotic prescription to reduce the use of antibiotics in preschool children with symptoms of URTIs. Nearly one-fourth of the respondents (23%) were not positive, while a third (30%) declared not having any opinion. The number of years in practice was significantly associated with the physician’s view on delayed antibiotic prescription. Physicians with more than 25 years of experience were less likely to be positive towards delayed antibiotic prescription compared to those with fewer years of experience (Table 2). Other factors (gender, practice location, and level of antibiotic use in the physician’s municipality) were not significantly associated with the views of the physicians.

**Table 2.** Number of years working in general practice associated with physicians’ positive views about delayed prescription.

	OR^a^	95%CI
0–5 years (*n* = 122)	1 (Reference)	
6–14 years (*n* = 168)	0.99	(0.62–1.61)
15–24 years (*n* = 162)	0.80	(0.50–1.30)
≥25 years (*n* = 115)	0.47	(0.27–0.83)

^a^Adjusted for gender, practice location, and level of antibiotic use in the physician’s municipality.

OR: odds ratio; 95%CI: 95% confidence interval.

### Physicians views on parents’ perceptions

*Willingness to wait-and-see.* Some physicians believe that most parents are willing to wait-and-see how the symptoms evolve and argue that a need to redeem the prescription is often prevented due to a remission of symptoms.

Parents will experience that the infection disappears spontaneously without treatment. (Physician K)

On the other hand, others argue that many parents would redeem the prescription and give antibiotics. They cite work pressure in parents and lack of babysitting possibilities as a possible explanation to this.

I believe many will redeem the prescription right away, both in the hope of the child getting better sooner and because they are under pressure from their job due to the child’s sick days. (Physician A)

*Need for reassurance.* Some physicians believe that delayed prescription with proper information is a reassurance to the parents because parents are given the responsibility to act if the symptoms persist or worsen.

The delayed antibiotic prescription gives the parents reassurance, making them more willing to wait and see when they have antibiotics on hand. (Physician J)

*Knowledge about infections and antibiotics.* Education and knowledge of parents were highlighted as the main factors for some parents supporting the use of delayed antibiotic prescriptions.

### Responsibility for treatment

Some physicians argue that a decision whether to give antibiotics can only be taken by a physician, not by the parents.

Parents are not able to assess whether there is a need for antibiotics. Instead of self-medication, they should come for a new consultation. (Physician M)

Other physicians argue that the strategy gives parents greater co-responsibility, making the parents more reflective about using antibiotics, and the strategy is referred to as a learning tool to educate the parents.

The parents are involved in the decision-making process and feel co-responsible for it. [The delayed antibiotic prescription has] an educational value—they have to take a critical stance, and they learn to have fewer expectations or demands for antibiotics. (Physician C)

### Structural factors

*Out-of-hours services.* Physicians argue that before weekends and holidays they are more inclined to use delayed prescriptions so that the parents can avoid out-of-hours visits, especially after the introduction of a new organization of the Capital Region of Denmark’s emergency departments, where nurses primarily treat patients.

Especially on Fridays, we experience that the threshold for giving antibiotics has dropped because we are not comfortable with the help our patients are offered by [the emergency department] ‘1813’ in the weekends. (Physician L)

### Workload

The physicians also emphasized a substantial workload as a reason for making them inclined to use delayed prescriptions, due to lack of time to follow up on the child.

## Discussion

### Main findings

Most (61%) of the respondents never or rarely used delayed antibiotic prescriptions for preschool children with URTIs. Most of them were positive (46%) rather than negative (23%) about the potential of delayed prescriptions to reduce antibiotic use. Physicians having no belief in the strategy were often those with significant work experience. Physicians with many years of experience may have a more paternalistic approach compared to their younger colleagues, or they could be more certain in the assessment of the child and, therefore, find the strategy less relevant. The physicians’ perception is influenced by the parents’ willingness to wait-and-see if the child recovers without antibiotic treatment. How both the physicians and parents consider the responsibility and capability of observation without antibiotic treatment and structural factors like out-of-hours services influence the physicians’ views about the strategy.

### Strengths and limitations of the study

Although many physicians responded (574), the response rate was low (49%). However, low response rates among GPs is a recognized problem in survey research [15,16]. No evidence of bias was found when comparing responding physicians with non-responding physicians on the following characteristics: type of physician (GP or trainee), gender, practice location, and level of antibiotic use in the physician’s municipality.

The responding physicians might have been more positive towards delayed antibiotic prescription or more engaged in the field of research and hence have a lower antibiotic use. However, out of the responding physicians, there were not more physicians from low-use municipalities, compared to the group of non-respondents (57% versus 59%).

Pretesting the questionnaire before distribution has reduced the risk of information bias in this study.

No information about the individual physician’s antibiotic use or prescribing style was available for our analyses.

### Comparison with existing literature

To the best of our knowledge, this is the first study focusing on delayed antibiotic prescription to children with URTIs. We found that physicians are mostly positive towards the strategy. A previous Norwegian study also found that most GPs (69%) use delayed antibiotic prescription as a reasonable strategy for patients with sinusitis and otitis, which are more common in children [17]. Consistent with our respondents, the Norwegian physicians emphasized shared decision-making and the opportunity to educate the patients.

In line with our study, a qualitative study from New Zealand found that delayed antibiotic prescription is a safety net that enables the patients to act when needed. The study also found that delayed prescription is a method to educate patients and empower them to be more involved in decision-making [18].

### Implications for future research and clinical practice

We found a discrepancy between the number of physicians with a positive attitude to delayed antibiotic prescription and the number of physicians using it. Delayed prescription has been shown to decrease inappropriate use of antibiotics, and measures should be put in place to facilitate the use of delayed antibiotic prescription in children with URTIs.

Some physicians stated that they became more positive towards the delayed prescription strategy since the introduction of a new organization of the emergency departments. This shows that the physician’s view of the quality of out-of-hours services is a significant factor, which should be considered when implementing the strategy.

Future studies should focus on the experiences of patients who receive a delayed prescription. Educated and knowledgeable parents are a logical target group for a delayed prescription, but there is a need for formalized recommendations regarding which patients are suitable for this strategy.

## Conclusions

Only 7% of the physicians often used a delayed prescription for children with URTIs, but most of them believe that delayed prescription for preschool children with URTIs could reduce antibiotic use. Physicians with many years of experience in general practice were less likely to be positive towards delayed antibiotic prescription compared to physicians with fewer years of experience. Different factors might influence the views of the physicians—from perceptions of parents to larger structural factors—towards implementing the strategy. However, the physician must consider if delayed antibiotic prescription is an appropriate strategy case by case.
